# Ischaemic giant Meckel’s diverticulum causing small bowel obstruction

**DOI:** 10.1093/jscr/rjaf904

**Published:** 2026-01-30

**Authors:** Andrew A V Bernstein, Ravi Aggarwal

**Affiliations:** General Surgery Department, Imperial College Healthcare NHS Trust, W21NY London, United Kingdom; Department of Surgery and Cancer, Imperial College London, W21NY London, United Kingdom

**Keywords:** surgery, Meckel’s diverticulum, management

## Abstract

Meckel’s diverticulum (MD) is the most common congenital abnormality of the small bowel, present in about 2% of the population. While typically asymptomatic, MD can cause complications such as small bowel obstruction (SBO), particularly in adults. Giant MD, defined as a diverticulum larger than 5 cm, is a rare cause of SBO and may lead to torsion, volvulus, or kinking of adjacent bowel. Preoperative diagnosis is often challenging as imaging findings are nonspecific, and MD may be misdiagnosed unless complications like inflammation or torsion occur. Surgical intervention, usually resection, is the treatment of choice for symptomatic MD, particularly in cases causing obstruction or containing ectopic mucosa. Although MD can be asymptomatic, the risk of complications in larger diverticula supports early surgical management in symptomatic cases to prevent severe outcomes like perforation or ischaemia. In this case report, SBO secondary to a giant MD requiring laparoscopic surgery is described.

## Introduction

Meckel’s diverticulum (MD) is the commonest congenital abnormality of the small bowel with many individuals remaining asymptomatic for their entire lives [1]. It is considered to be a true diverticulum containing all layers of the small bowel which usually forms in the fifth week of foetal development when there is failure of the vitelline duct to obliterate [1]. MD is described in terms of the ‘rule of twos’ with it occurring in 2% of the population, occurring ~2 feet from the ileo-caecal valve and twice more common in men than women [2]. When symptomatic, one of the most common presentations is painless rectal bleeding; however, the literature suggests vomiting, abdominal pain, and intestinal obstruction are now more common [3].

In the adult population, obstruction is the most common presenting symptom [4]. In 2014, Akbulut *et al*. [5], described a rare case of acute small bowel obstruction (SBO) secondary to a giant MD which resulted in axial torsion and a small bowel resection with ileal-ileal anastomosis. Cartense *et al*. further [6] described a case of acute bowel obstruction secondary to a giant MD in 2011 which led to acute small bowel intestinal obstruction, requiring a laparotomy and diverticulectomy.

In this case report, we describe a further rare complication of a giant MD leading to acute SBO treated laparoscopically.

## Case report

A 41-year-old gentleman, with no known comorbidities and no previous abdominal surgeries, was referred to our emergency department with a 1 day history of feeling generally unwell with associated abdominal pain. He described the abdominal pain as central and intermittent in nature. He reported to have started vomiting on the day prior to his admission which had progressively become more severe. Furthermore, he had not opened bowels or passed flatus over the last day.

On initial presentation, he was hypertensive with a blood pressure of 184/110, his heart rate was 95 with a temperature of 36°C.

His abdominal examination demonstrated right upper quadrant tenderness, with no generalized peritonism.

His laboratory investigations demonstrated a white cell count of 16.4, haemoglobin (Hb) of 183 with a C-reactive protein of <1. His lactate was initially 2.7 and after resuscitation, it improved to 1.8. Both his liver function tests and renal function tests were normal.

**Figure 1:**
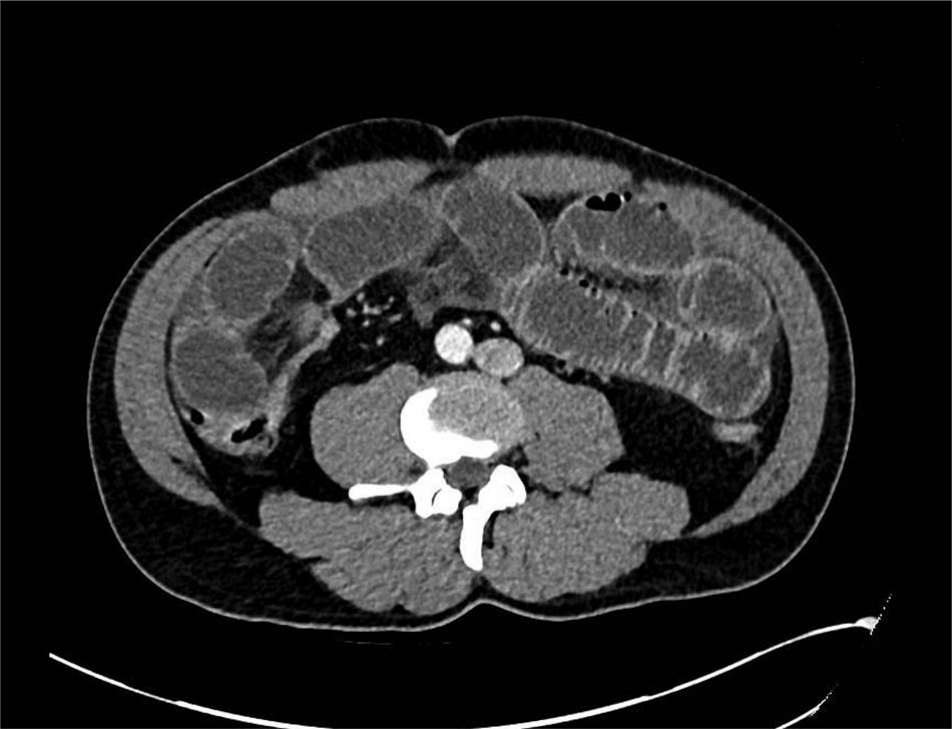
CT showing transition point in right iliac fossa.

**Figure 2:**
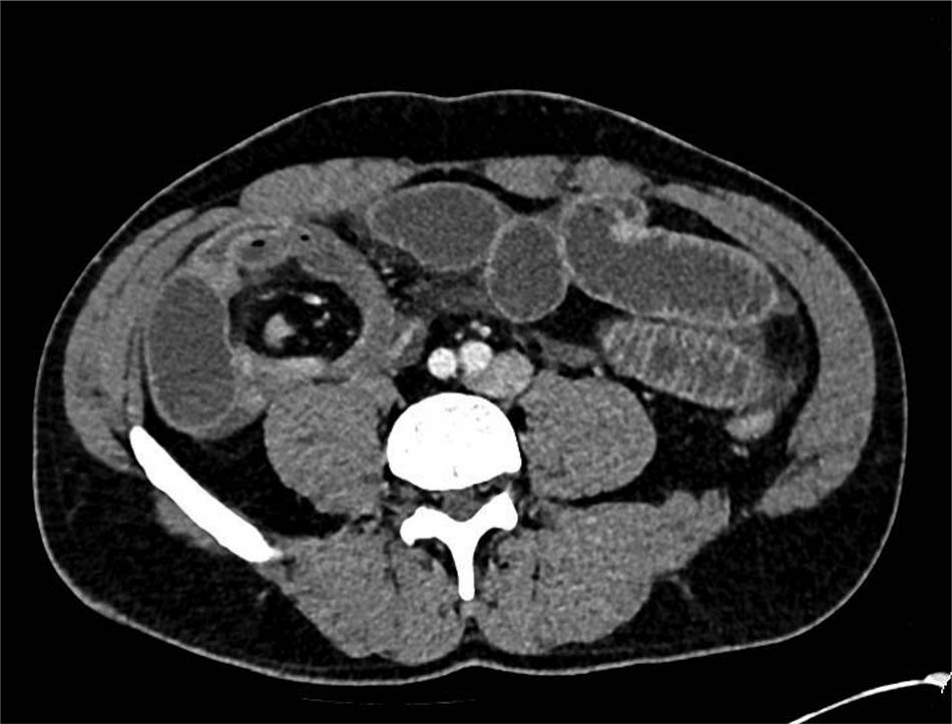
CT showing abnormality in right iliac fossa with dilated bowel loops.

A computed tomography (CT) scan was performed which showed: ‘The findings raise the possibility of a closed loop SBO involving the ileal small bowel in the right iliac fossa. There is a slightly thickened segment of ileum with mildly reduced enhancement with a transition point proximally and distally identified. There is no evidence of perforation currently’ (Figs 1 and 2).

A nasogastric tube was inserted which drained 400 ml over bilious fluid and the patient was treated conservatively overnight.

The CT scan was subsequently discussed in a multidisciplinary team meeting the following day which again demonstrated features of SBO with a clear transition point in the distal ileum. There was no mention of a possible MD being the culprit.

**Figure 3:**
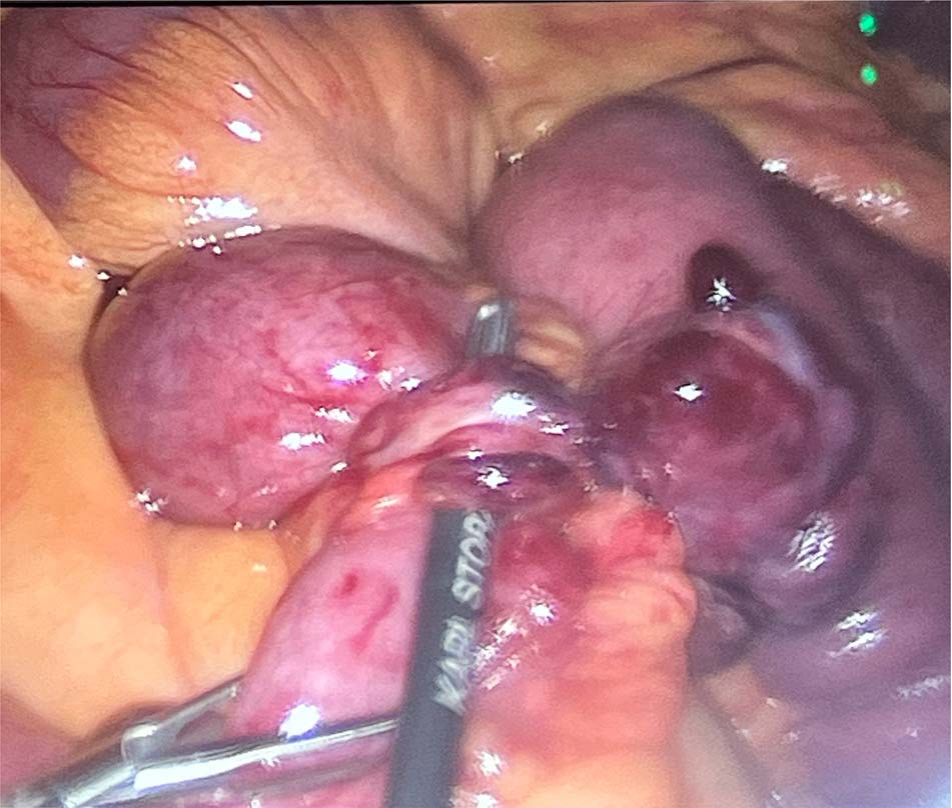
Adhesions from the tip of the giant Meckel’s diverticulum.

**Figure 4:**
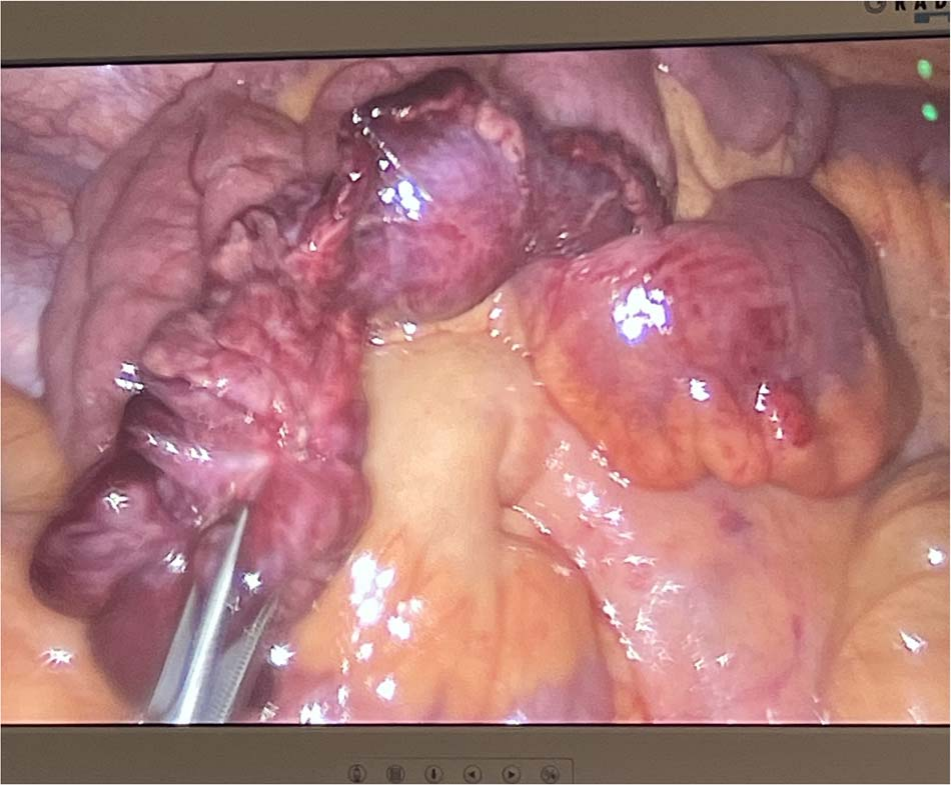
Ischaemic giant Meckel’s diverticulum.

**Figure 5:**
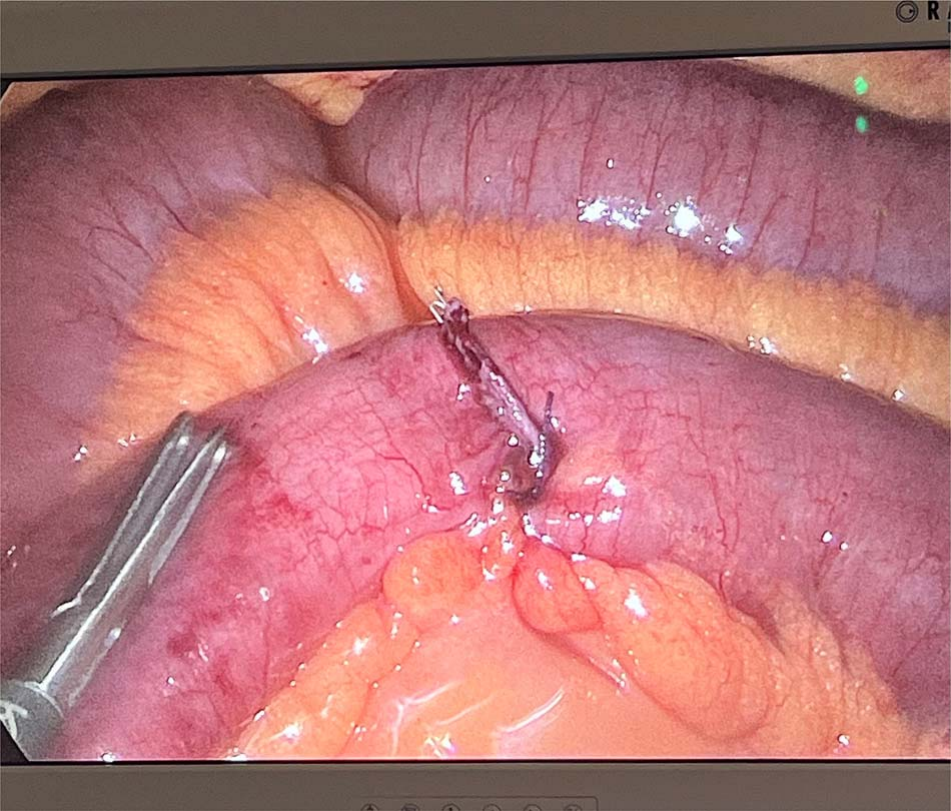
Terminal ileum post-resection of giant Meckel’s diverticulum.

He was taken to theatre for a diagnostic laparoscopy. Intraoperatively, it was noted there was a long, thin ischaemic MD torting around the distal ileum causing obstruction of the proximal small bowel. He underwent adhesiolysis of a band at the tip of the diverticulum (Fig. 3) which then allowed for detorsion of the diverticulum around the terminal ileum (Fig. 4). The MD was subsequently stapled off and excised (Fig. 5). The rest of the small bowel was pink and preinstalling well.

Post-operative histology showed a ‘pouch like structure with single staple line and blind ended measuring 95 mm in length by up to 22 mm in diameter. The serosa appears dusky with microscopic histology confirming early ischaemic change’, which was in keeping with the intra-operative diagnosis.

The patient recovered well and was discharged 3 days after the procedure with no complications.

## Discussion

MD is the most common congenital anomaly of the gastrointestinal tract, present in ~2% of the population [1]. Despite this prevalence, it remains largely asymptomatic, with complications occurring in only a minority of individuals—most frequently in children [3]. In adults, however, MD is an important cause of SBO with obstructions of various types being the most common presenting symptom, occurring in almost 40% of patients [7]. This case illustrates a rare presentation of SBO secondary to a giant MD, a condition that presented diagnostic and surgical challenges.

The pathophysiology of obstruction in MD can vary. It may result from volvulus around a fibrous band, intussusception with the MD as a lead point or internal herniation [8]. In this case, the obstruction was to a giant MD—defined as one larger than 5 cm—which can act as a fixed segment and precipitate torsion or kinking of adjacent bowel [9]. The unusual size of the diverticulum played a direct mechanical role in the obstruction with it torting around the distal ileum. Giant MDs are exceedingly rare and have been associated with a higher risk of complications, including perforation and inflammation [5, 10].

Pre-operative diagnosis of symptomatic MD remains challenging. Imaging findings are often nonspecific, particularly when the diverticulum lacks ectopic mucosa or is not inflamed and it may be mistaken for small bowel [11]. While CT is the modality of choice for SBO, MD is frequently misidentified or overlooked unless complications such as volvulus, intussusception, or localized inflammation are present and even then, it is still a difficult pre-operative diagnosis [12]. In our case, although imaging identified the transition point, the diagnosis of MD was ultimately confirmed intra-operatively. This supports findings in the literature suggesting that definitive diagnosis often relies on surgical exploration, particularly in patients with virgin abdomens and no prior history of surgery or hernia [13].

Management of symptomatic MD is surgical. Resection is advised for diverticula causing obstruction or containing ectopic mucosa, and segmental bowel resection may be necessary when there is involvement of adjacent ileum or compromised viability [14]. In our patient, the giant MD necessitated adhesiolysis of a band and resection of the diverticulum to relieve the obstruction and ensure resolution of the bowel obstruction, which was done laparoscopically.

This case reinforces the importance of considering MD as a possible and significant cause of SBO in adults, particularly in the absence of more common aetiologies. Early surgical intervention in symptomatic MD remains critical to avoid complications such as ischaemia or perforation [14], however in asymptomatic MD, resection remains controversial with one study suggesting 800 resections need to be made to save a life which could lead to significant post-operative complications [15].
